# Supplementary material for: An Introduction to Cystoscopy for OB/GYN Residents
Source: MedEdPORTAL. 2022 Feb 7;18:11220. doi: 10.15766/mep_2374-8265.11220 (PMC8818811; doi:10.15766/mep_2374-8265.11220)
Supplement: Supplementary file 1 — Instructors Guide.docxStation Details.docxCourse Checklist.docxEvaluation Forms.docx [file mep_2374-8265.11220-s001.zip › C. Course Checklist.docx]

| **Course Checklist** | | | |
| --- | --- | --- | --- |
| Trainee Name: ___________________________________________________________________ | | | |
| Preceptor Name: ___________________________________________________________________ | | | |
|  |  |  |  |
| Pre-course Checklist | | Post-course Checklist | |
|  |  |  |  |
| Identify the Following: | | Identify the Following: | |
|  | Sheath size |  | Sheath size |
|  | Channel size |  | Channel size |
|  | Lens and degree |  | Lens and degree |
|  | Bridge |  | Bridge |
|  | Camera |  | Camera |
|  | Water tubing/stop cock |  | Water tubing/stop cock |
|  | Light cord |  | Light cord |
|  | Working ports/nipple |  | Working ports/nipple |
|  |  |  |  |
| Put Together the Following: | | Put Together the Following: | |
|  | Sheath |  | Sheath |
|  | Lens 30 degree |  | Lens 30 degree |
|  | Bridge |  | Bridge |
|  | Camera |  | Camera |
|  | Water tubing/stop cock |  | Water tubing/stop cock |
|  | Light cord |  | Light cord |
|  |  |  |  |
| Steps of the Procedure: | | Steps of the Procedure: | |
|  | Equipment assembly |  | Equipment assembly |
|  | Fluid type |  | Fluid type |
|  | White balance |  | White balance |
|  | Lubrication |  | Lubrication |
|  | Insertion of scope |  | Insertion of scope |
|  | Visualization of bladder |  | Visualization of bladder |
|  |  |  |  |
|  |  |  |  |
|  |  |  |  |
