# Supplementary material for: An Introduction to Cystoscopy for OB/GYN Residents
Source: MedEdPORTAL. 2022 Feb 7;18:11220. doi: 10.15766/mep_2374-8265.11220 (PMC8818811; doi:10.15766/mep_2374-8265.11220)
Supplement: Supplementary file 1 — Instructors Guide.docxStation Details.docxCourse Checklist.docxEvaluation Forms.docx [file mep_2374-8265.11220-s001.zip › D. Evaluation Forms.docx]

**Pre-Course** Evaluation by OB/Gyn Resident – 2020

Resident Name__________________ PGY Level __________

Rate your knowledge of cystoscopy equipment prior to taking this course:__________

1 I have no knowledge of equipment

2 I can recognize equipment, but can’t put it together

3 I can recognize equipment and put it together with assistance

4 I can recognize equipment and put it together without assistance

5 I can recognize equipment, put it together without assistance and trouble shoot problems

Rate your anxiety level with the procedure of cystoscopy prior to taking this course:__________

1 High anxiety

2 Moderate anxiety

3 Some anxiety – Normal

4 Minimal anxiety

5 No anxiety

**Post-Course** Evaluation by OB/Gyn Resident – 2020

Resident Name____________________ PGY Level __________

Rate your knowledge of cystoscopy equipment after taking this course:__________

1 I have no knowledge of equipment

2 I can recognize equipment, but can’t put it together

3 I can recognize equipment and put it together with assistance

4 I can recognize equipment and put it together without assistance

5 I can recognize equipment, put it together without assistance and trouble shoot problems

Rate your anxiety level with the procedure of cystoscopy after taking this course:__________

1 High anxiety

2 Moderate anxiety

3 Some anxiety – Normal

4 Minimal anxiety

5 No anxiety

Use the following scale to evaluate the course overall:__________

1 No knowledge/skill gained during course

2 Minimal knowledge/skill gained during the course – Some gain, but not ready for my first case

3 Some knowledge/skill gained during the course – Moderate gain, but not ready for my first case

4 Adequate knowledge/skill gained – I am ready for my first cysto case

5 Superior knowledge/skill gained – I am ready to graduate and perform cysto on my own

Any part of the course which was completely useless?

Any part of the course which was absolutely essential?

Any suggestions for additions to the course?
