# Supplementary material for: An Introduction to Cystoscopy for OB/GYN Residents
Source: MedEdPORTAL. 2022 Feb 7;18:11220. doi: 10.15766/mep_2374-8265.11220 (PMC8818811; doi:10.15766/mep_2374-8265.11220)
Supplement: Supplementary file 1 — Instructors Guide.docxStation Details.docxCourse Checklist.docxEvaluation Forms.docx [file mep_2374-8265.11220-s001.zip › A. Instructors Guide.docx]

**An Introduction to Cystoscopy for OBGYN Residents: Instructors Guide**

**Educational Objectives**

By the end of the workshop, learners will:

1. Identify each piece of equipment used in cystoscopy
2. Assemble cystoscopy equipment independently
3. Decrease feelings of anxiety surrounding performing cystoscopy as measured by pre- and post-course surveys

**Purpose of Activity:**

With work hour constraints there is an increased emphasis on finding ways to educate residents as efficiently as possible. *An Introduction to Cystoscopy for OBGYN Residents* was developed to give OBGYN trainees a chance to work with cystoscopy equipment outside the operating room in hopes that decreased anxiety around cystoscopy and increased comfort with the equipment would lead to better educational experiences in future cystoscopic cases. This workshop is meant to take place over the course of roughly one hour and includes two stations: one focused on familiarity with assembly and disassembly of the cystoscope and another allowing for simulation of cystoscopy using a pig bladder.

**Terminology:**

Instructor: Urology or urogynecology faculty member organizing, facilitating and managing the workshop

Preceptor: Urology or urogynecology faculty or senior urology resident leading a station

**Resources included and how to use each file:**

- - - 1. Instructor’s Guide: to be consulted by the instructor prior to and during the workshop. Contains list of resources, practical implementation advice, and schedule for the day of the workshop.
      2. Station Details: to be used by instructor and preceptors during preparation for and setup of the workshop, can be referenced by preceptors during the workshop to make sure material is properly covered. Contains necessary supplies for the two stations, how to set up each station, and describes the procedure for each station.
      3. Course checklist: to be used by preceptors to record activities performed by participants at each station pre- and post-instruction.
      4. Evaluation forms: pre- and post-course evaluation forms to be completed by participants.

**Practical advice for implementation:**

Prepare all forms needed for preceptors and participants prior to the workshop. Make sure to have an adequate amount of time on the day of the activity to set up each station and that each station has the necessary supplies. It is also helpful to have extra pieces of equipment if possible, to have replacements readily available should equipment malfunction. Each station is designed to take place over a period of about 20 minutes. Stations do not need to be completed in a specific order, allowing for all stations to be used at one time. Multiple rounds of residents can be run through the activity in one day if needed, depending on the size of the group and the number of duplicate stations that can be set up.

**Timeline:**

1. Distribute blank course checklist forms to preceptors.
2. Review procedures of each station with preceptors, verify that equipment is functioning correctly at each station, make sure preceptors are aware of where to find replacement equipment if needed.
3. Distribute pre-course evaluations to residents and have them complete the evaluations.
4. Begin workshop:
   1. 10-15 minutes: Introduce educational objectives, have participants view “Cystoscopic Findings: a Video Tutorial”^1^. Answer any questions that participants have after viewing the video. This video can be found online by visiting the home page for PubMed, searching “Cystoscopic findings: a video tutorial”, following the link to the *Springer* website and scrolling to the electronic supplementary materials section.
   2. 20 minutes: Participants divide into groups of 2-3 and complete one of the two stations
   3. 20 minutes: Participants switch stations and complete their second station

*If multiple rounds of participants are to complete the workshop in the same day the next group should begin at a above when the preceding group has finished the first station and is progressing to c

1. After participants have completed both stations, have residents complete the post-course evaluation form.
2. Note what went well during the workshop and what could have been better executed. Review feedback from evaluation forms to improve subsequent implementations.

**References**

1. Lenherr SM, Crosby EC, Cameron AP. Cystoscopic findings: a video tutorial. *Int Urogynecol J*. 2015;26(6):921-923. doi:10.1007/s00192-014-2614-4
