# Supplementary material for: An Introduction to Cystoscopy for OB/GYN Residents
Source: MedEdPORTAL. 2022 Feb 7;18:11220. doi: 10.15766/mep_2374-8265.11220 (PMC8818811; doi:10.15766/mep_2374-8265.11220)
Supplement: Supplementary file 1 — Instructors Guide.docxStation Details.docxCourse Checklist.docxEvaluation Forms.docx [file mep_2374-8265.11220-s001.zip › B. Station Details.docx]

**An Introduction to Cystoscopy for OBGYN Residents: Station Details**

**Educational Objectives**

By the end of the workshop, learners will:

1. Identify each piece of equipment used in cystoscopy
2. Assemble cystoscopy equipment independently
3. Decrease feelings of anxiety surrounding performing cystoscopy as measured by pre- and post-course surveys

**Station 1: Cystoscopy Equipment and Assembly**

Station Supplies:

- Table
- Sheath and obturator: 17F and 22F
- Bridge
- Lens: 0, 30 and 70 degree
- Irrigation tubing and adaptor
- Light cord
- Camera
- Nipples
- Biopsy forceps

Station Setup

Arrange cystoscopy equipment listed above on a table. Group sheaths, obturators, bridge and lenses on one side of the table. Irrigation tubing, light cord, camera, nipples, and biopsy forceps should be grouped on the other side of the table.

Station Procedure

The preceptor should start the station by asking participants to name each piece of equipment on the table, and by marking on the pre-course checklist the equipment which the learner could name successfully. Next the preceptor should review all of the equipment names and their respective functions with participants. Afterwards, the preceptors should have participants attempt to assemble the scope, and mark which equipment the learners can successfully put together on the pre-course checklist. Then preceptors should demonstrate how to assemble each piece of equipment and go over the following:

- The necessity of proper personal protective equipment (PPE) including face mask, gown, gloves, and eye protection
- Light cord safety and the fire/burn risk that the light cord poses
- Assembly of the lens, bridge, and sheath
- Connecting the light cord, camera, irrigation tubing and nipple
- How to white balance the camera
- The need for lubrication of the scope
- The need for a nipple if a working port is to be used
- Proper handling of the scope to minimize risk of damaging the equipment
- Irrigation fluid type, noting that saline is used when cytology is needed, and that water should be used if electrocautery may be necessary

Participants should then be given the opportunity to assemble and disassemble the cystoscope multiple times until they become comfortable with the process. Preceptors should answer any questions the participants have about the equipment or how it should be assembled. To wrap the station up participants should again name the equipment and assemble the scope. Preceptors should mark on the post-course checklist the items that are successfully named and the items that are successfully assembled.

**Station 2: Cystoscopy Simulation**

Station Supplies

- Table
- Sheath 22F
- Bridge
- Lens 30 degree
- Irrigation tubing and adaptor
- 1L bags normal saline or sterile water
- Light cord
- Camera
- Storz Tele Pack or other monitor/light source
- Biopsy forceps
- 4x4 gauze
- IV pole
- Male pig bladder
- Basin
- Wire baking rack or other grate apparatus
- 2-0 or 3-0 suture (any type)
- Scissors or scalpel

Station Setup

*Pig bladder preparation*

Evaluate the available pig bladders, it is recommended that more bladders are requested than are necessary to have redundancy should one become damaged during use. Select the largest bladders of those that you receive to use during the exercise. Remove the majority of the urethra, leaving only a small stump (pictured) by cutting through the tissue with a scalpel or scissors. Remove excess fat/soft tissue from the bladder by gently pulling the tissue away with a gloved hand.

author owned


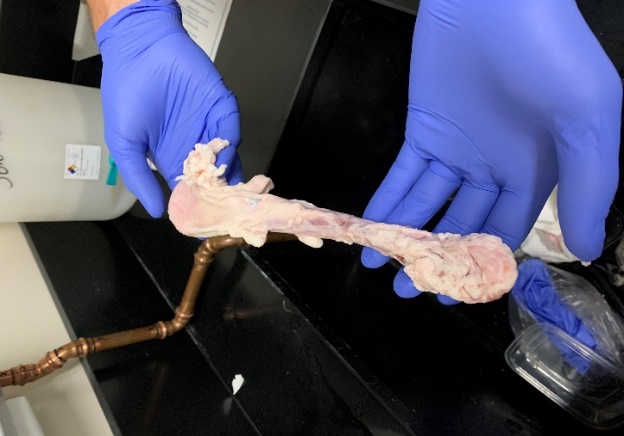


The bladder should then be attached to a grate with 2-0 or 3-0 suture using one stitch in the midline at the bladder neck and a single stitch on either side of the bladder, closer to the bladder dome. These stitches should be superficial enough to not violate the bladder lumen to avoid leakage of irrigation, but deep enough to provide a stable attachment. Stability should be assessed by inserting a cystoscope into the urethra and evaluating how securely the bladder is attached. Irrigation can be trialed at this point to see if the bladder will fill without excessive leakage of irrigation.


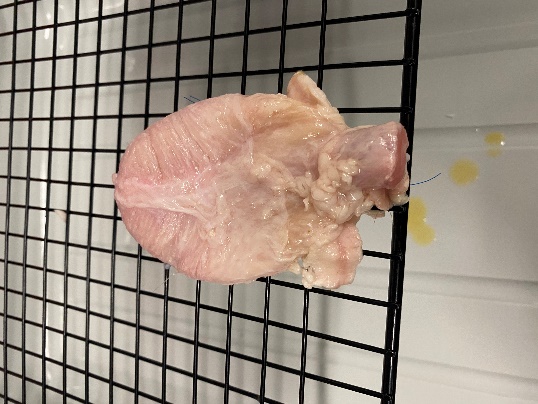


author owned

*Assembly of the simulation apparatus*

Place the grate and bladder over a basin and set on the center of a table. The basin will allow for collection of irrigation fluid during the activity. Fully assemble the cystoscope, place one nipple over a working channel and attach the camera, light cable and irrigation tubing to the scope. Place the monitor somewhere that can be easily seen by participants, and hang the irrigation fluid on an IV pole. Ensure that multiple bags of irrigation fluid are available for each station. Test the light source and monitor to verify that the picture quality is adequate for participants. Once set up is complete have a preceptor perform cystoscopy on the pig bladder to ensure all components are functioning properly and the station is ready for the workshop to begin. One example of this station setup is pictured below, but can be modified depending on the equipment and space available. After testing is complete turn off light, remove the light cord, irrigation tubing, and camera from the scope and set them on table adjacent to the assembled cystoscope.


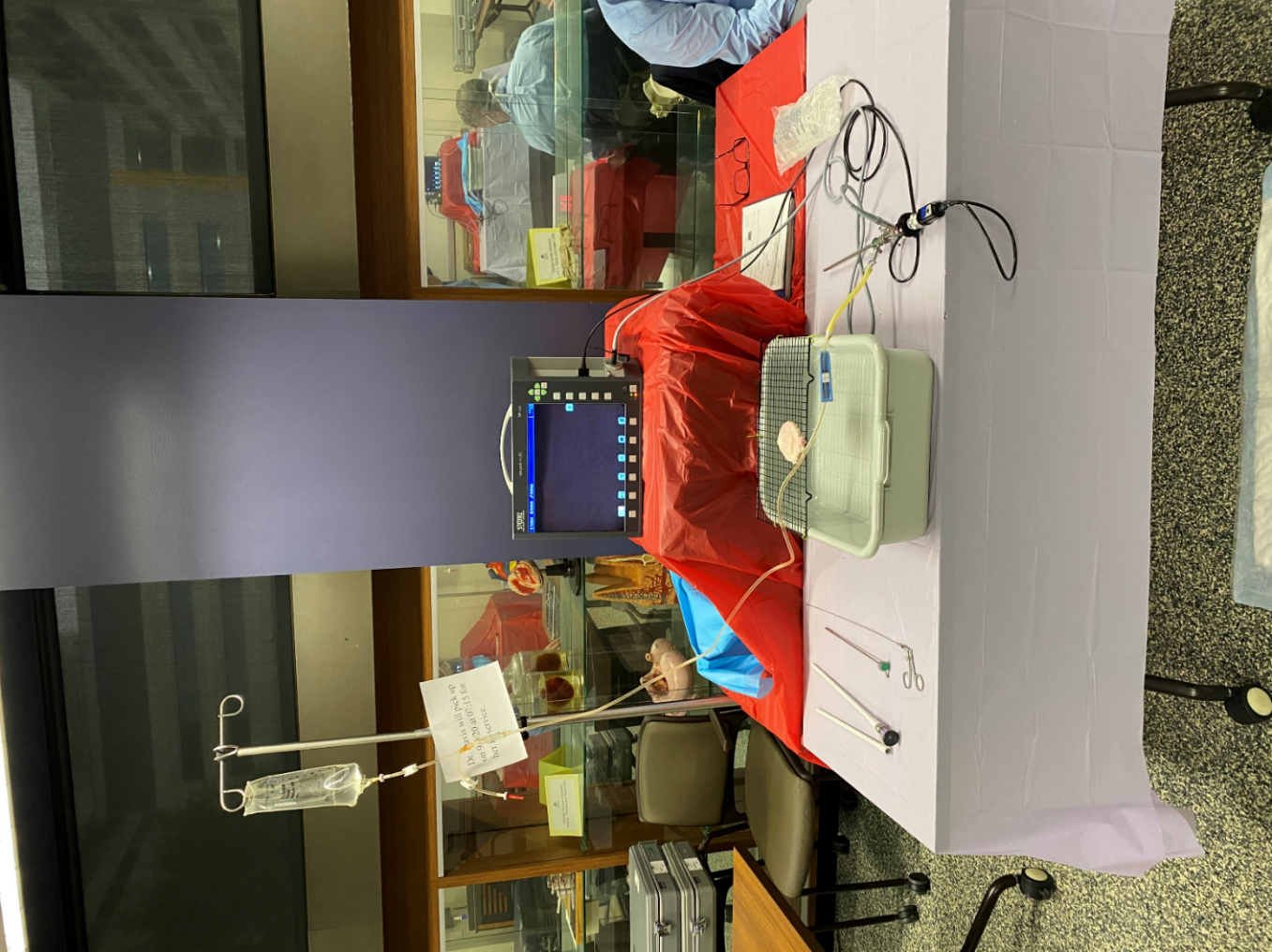


author owned

Station Procedure

The preceptor should start by asking participants to list the steps of performing cystoscopy and by marking on the pre-course checklist the steps that the participant names. At that point the preceptor should review the following steps and information:

- Verify correct assembly of the scope, nipple placement, and closure of working channels/outflow port
- Connection of the light cord, irrigation tubing, and camera. Again emphasize the fire/burn risk the light cord poses when not connected to the scope
- White balancing of the scope, adjusting the focus of the scope
- Lubrication of the scope
- Insertion of the scope and the importance of keeping the lumen of the urethra in the center of the image while inserting
- The need to turn inflow off once bladder is sufficiently distended for visualization. The importance of managing inflow and outflow to avoid excessive bladder distension
- Bladder visualization: that there are multiple systems for visualizing the entire bladder and that participants should have a pattern in mind that they use each time they perform cystoscopy
- The process of breaking the scope to drain the bladder, turning inflow on, re-attaching the scope and filling the bladder to continue with procedure. Emphasize that this can be useful when visualization is an issue, especially at the beginning of a procedure if a patient’s bladder is full of urine
- The importance emptying the patient’s bladder at the end of the case to avoid leaving patient with a distended bladder

Participants should then be given the opportunity to perform cystoscopy with coaching from the preceptor. They should start by attaching the light cord, camera and irrigation tubing and then proceed through the procedure. This can be repeated until participants feel comfortable. If there is extra time participants can practice inserting a biopsy forceps through the scope and manipulating them within the bladder. The biopsy forceps should not be used on the pig bladder to prevent damage that would inhibit further use of the station. To wrap the station up participants should again proceed through simulated cystoscopy without coaching. Preceptors should mark on the post-course checklist the steps that are successfully performed.
