# Supplementary material for: An Introduction to Cystoscopy for OB/GYN Residents
Source: MedEdPORTAL. 2022 Feb 7;18:11220. doi: 10.15766/mep_2374-8265.11220 (PMC8818811; doi:10.15766/mep_2374-8265.11220)
Supplement: Supplementary file 1 — Instructors Guide.docxStation Details.docxCourse Checklist.docxEvaluation Forms.docx [file mep_2374-8265.11220-s001.zip › Educational_Summary_Report_11220.pdf]

MedEdPORTAL® The AAMC Journal of Teaching and Learning Resources

Original Publication Open Access

An Introduction to Cystoscopy for OB/GYN Residents

Nayan Shah, MD*, Robert Medairos, MD, Sumana Koduri, MD, Carley Davis, MD

*Corresponding author: nayshah@mcw.edu

Abstract

Introduction: OB/GYN residents' preparedness to perform cystoscopy after residency may vary as the ACGME requires only 10 cystoscopic cases to be performed during training. Given residents' potentially limited exposure to cystoscopy, supplemental educational activities centered around increasing familiarity with the procedure may be useful. The objective of this workshop was to provide an opportunity for OB/GYN residents to become more comfortable with cystoscopic equipment and performing cystoscopy. **Methods:** We showed a video of common pathology seen on cystoscopy and then progressed through two hands-on stations. One station focused on equipment familiarity, with learners identifying equipment and then practicing assembling and disassembling the cystoscope. The other station allowed for simulated cystoscopy utilizing a pig bladder. We used a checklist assessment and pre- and postcourse surveys to evaluate familiarity with equipment and anxiety surrounding performing cystoscopy. **Results:** Twenty residents ranging from PGY 1s to PGY 4s who participated in this workshop over the past 2 years completed both pre- and postcourse evaluations. There was statistically significant improvement in ratings of familiarity with equipment and anxiety surrounding the procedure. All participants whom we assessed showed improvement in identifying and assembling equipment as well as in performing the steps of the procedure independently. **Discussion:** This workshop provided OB/GYN residents with an opportunity for hands-on cystoscopic experience. Through direct assessment and evaluation forms, the workshop was shown to be a beneficial activity for improving cystoscopic knowledge.

Keywords
Cystoscopy, OB/GYN, Clinical/Procedural Skills Training

Educational Objectives

By the end of this workshop, learners will be able to:

1. Identify each piece of equipment used in cystoscopy.
2. Assemble cystoscopy equipment independently.
3. Decrease feelings of anxiety surrounding performing cystoscopy, as measured by pre- and postcourse surveys.

Introduction

In the era of work hour restrictions, there is increased pressure for resident teaching that can maximize resident benefit over short time periods. In obstetrics and gynecology (OB/GYN), cystoscopy is a useful skill that some trainees may have limited exposure to. The ACGME requires 10 cystoscopic cases for OB/GYN residents.[1] With potentially limited exposure to cystoscopic cases in clinical training, didactic and simulated educational sessions centered around cystoscopy may be useful for OB/GYN residency programs.

A recent study evaluated OB/GYN residents' preparedness for female pelvic medicine and reconstructive surgery (FPMRS) fellowships following residency training. FPMRS senior fellows and program directors felt that incoming fellows were not proficient at performing cystoscopy, and there was widespread concern that OB/GYN residents' cystoscopy experience was below expectations.[2] While cystoscopy is especially important for future FPMRS fellows, it is a useful skill for many OB/GYN practitioners as it can be used to verify ureteral patency and monitor for iatrogenic bladder injuries during gynecologic procedures. Our aim for this workshop was to increase familiarity with cystoscopy equipment and to provide an opportunity for trainees to practice cystoscopy skills so they could feel less anxiety in future cystoscopic procedures.

The workshop was modeled after "Introduction to Endoscopy for Urology Residents," an activity previously published in *MedEdPORTAL* in which incoming PGY 2 urology residents

Citation:
Shah N, Medairos R, Koduri S, Davis C. An introduction to cystoscopy for OB/GYN residents. *MedEdPORTAL*. 2022;18:11220.
https://doi.org/10.15766/mep_2374-8265.11220

Copyright © 2022 Shah et al. This is an open-access publication distributed under the terms of the Creative Commons Attribution license.

were rotated through stations that allowed them to become more familiar with endoscopy equipment commonly used in urologic procedures.[3] These stations focused on developing familiarity with equipment setup and use. They utilized rapid cycle deliberate practice, an educational tool where learners cycle between deliberate practice and immediate feedback to rapidly improve, to teach residents how to use various pieces of urologic equipment. That same strategy was used in our workshop as learners were able to practice skills numerous times in front of proctors who could help them immediately improve. There are no *MedEdPORTAL* publications centered on teaching OB/GYN residents cystoscopy. Bowling and colleagues showed that a 2-hour didactic session using a balloon model to practice cystoscopy increased cystoscopic skill and knowledge for OB/GYN residents.[4] It has also been shown that a weekly course over 6 weeks focused on cystoscopy simulation can be beneficial for teaching OB/GYN residents cystoscopy.[5] Our program takes place over 1 hour, providing an option for increasing cystoscopic knowledge within a short time period. We utilize a pig bladder for our cystoscopic simulation, offering another low-cost alternative to a balloon model. Furthermore, we present a detailed curriculum that can be easily replicated at other institutions to enhance the cystoscopic skills of their OB/GYN trainees.

Methods

Our workshop consisted of viewing of a preliminary video showing potential pathology that could be identified during cystoscopy, followed by two hands-on stations. The session took place over roughly 60 minutes so that it could occur in the morning before subsequent cases during scheduled didactic time the same day. Residents completed both pre- and postworkshop surveys that evaluated knowledge of cystoscopy equipment and anxiety around performing cystoscopy on patients. During the first 10-15 minutes of the activity, we introduced our educational objectives and showed a video on common pathology seen on cystoscopy. Trainees watched the video together as a large group and had the opportunity to ask questions after viewing. The residents then progressed through the two stations outlined below in groups of two to three people. Each station took about 20 minutes. Our workshop accommodated six to nine participants at a time as we had three sets of the two stations. Larger groups could be accommodated if more instructors and stations were available. More details on the time line and execution of the activity can be seen in Appendix A. We obtained institutional review board (IRB) approval to evaluate the effectiveness of our workshop from the Medical College of Wisconsin IRB.

Introductory Video

We played an initial video that was produced by the University of Michigan urology and OB/GYN departments in 2015.[6] This video can be found using a PubMed search and the title given in the References, below. The video displayed pathology representing potential incidental findings on cystoscopy and detailed which findings could be safely observed as opposed to which pathology merited further intervention. The video showed both benign and malignant pathologies. Benign cystoscopic findings included squamous metaplasia, duplicated ureteral orifice, ureterocele, Hutch diverticulum, bladder trabeculation, urachal cyst, interstitial cystitis with and without Hunner's lesion, endometriosis in the bladder, port-wine stain due to Klippel-Trénaunay-Weber syndrome, nephrogenic (mesonephric) metaplasia, and cystitis glandularis (intestinal metaplasia). The malignant pathological conditions covered in the video included papillary urothelial neoplasm of low malignant potential, carcinoma in situ, high-grade urothelial carcinoma, and urachal cancer. Edema from ureteral stents and stone-encrusted mesh were also shown in the video.

Stations

Equipment review: One station centered on identifying and putting together cystoscopy equipment. First, we presented various sheaths (17F, 22F), bridges, obturators, lenses (0°, 30°, 70°, 120°), and other equipment needed for cystoscopy to our participants. We laid out the equipment on a table so that it was easily accessible for the trainees. Instructors reviewed the name of each piece of equipment with learners and went over how the equipment functioned. Next, instructors demonstrated proper assembly and disassembly of the cystoscope. This process included connecting the light cord, camera, and water tubing to the scope. A complete list of equipment and processes reviewed with learners can be seen in Appendix B. After this initial phase, we asked learners to identify each piece of equipment from a list provided to the preceptor. The checklist used by the preceptors can be seen in Appendix C. If the trainees could not identify a piece of equipment, then it was reviewed again with them. Next, we coached participants through putting equipment together themselves and repeated the process until they felt comfortable with assembling and disassembling the cystoscope. The residents then demonstrated their ability in assembling and disassembling the cystoscope without assistance.

Cystoscopy simulation: Another station provided a simulated cystoscopy experience in which participants performed rigid cystoscopy on male pig bladders. The pig bladders were relatively inexpensive, costing about $5 per bladder. Of the

Copyright © 2022 Shah et al. This is an open-access publication distributed under the terms of the Creative Commons Attribution license.

pig bladders that we obtained for this activity, we selected the largest to be used in our stations. We prepared the pig bladders by removing excess fat and soft tissue as well as most of the urethra. Figure 1A shows where the urethra was cut; we left only a small stump of urethra on the bladder. This allowed for a more optimal simulation as the urethra was fragile and more difficult to secure for repeated use. We secured the bladder to a metal grate using one stitch in the midline at the bladder neck and a single stitch on either side of the bladder, closer to the bladder dome. The attachment of the bladder to the metal grate is shown in Figure 1B. These stitches were superficial enough to not violate the bladder lumen in order to avoid leakage of irrigation but deep enough to provide a stable attachment. We placed the grate and bladder above a basin so that any excess irrigation would be contained. We placed the monitor and light source above the grate apparatus so it could be easily visualized by participants. Prior to the workshop, we tested each bladder to make sure that it could be filled and that it would expand without significant leakage. Figure 2 shows the complete setup for this station.

We preassembled the cystoscope with light cord, irrigation, and camera all connected prior to trainees starting the station. We used two 1-liter bags of saline irrigation fluid at each station during our workshop, and more were readily available. Further details about this station's assembly can be found in Appendix B. We coached residents on proper grip of the cystoscope and gave them the opportunity to perform basic cystoscopy skills, including white balancing the scope, filling the bladder, visualizing the bladder, and draining the bladder by breaking the scope. Instructors emphasized having a system to visualize the entirety of the bladder and reviewed techniques for doing so with the trainees.

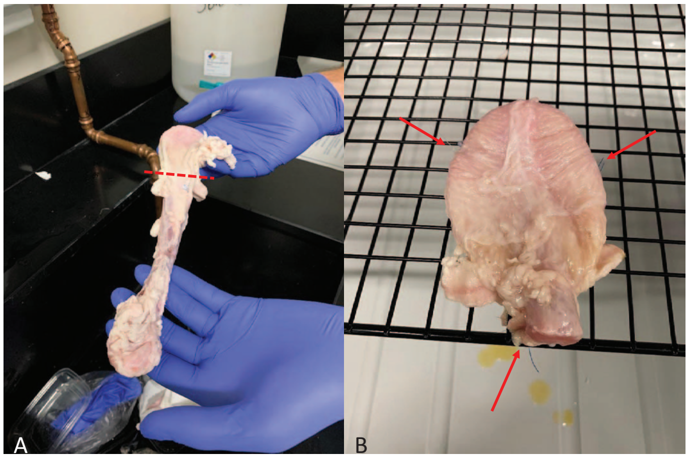

Figure 1. Preparation of pig bladder for simulated cystoscopy. A: Dashed line depicts level at which urethra was transected and removed. B: Arrows depict where sutures were placed to secure pig bladder to grate. Photographs are author owned.

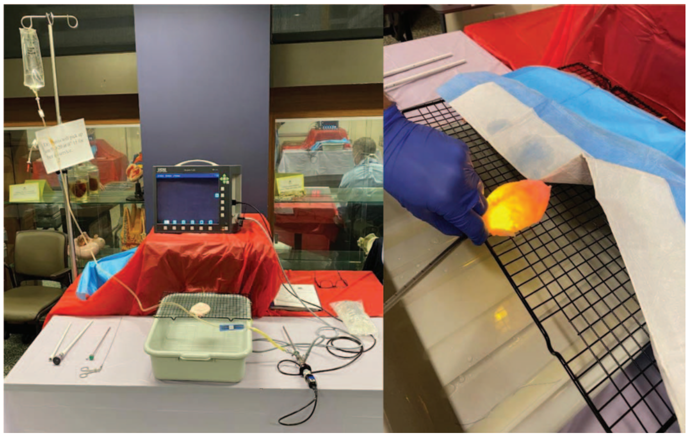

Figure 2. Simulated cystoscopy station setup. Left: Complete station setup for simulated cystoscopy experience with pig bladder. Right: Pig bladder with cystoscope inserted and in use. Photographs are author owned.

Evaluation

We kept evaluation forms for the activity short to minimize the amount of time residents would need to spend on them. The precourse evaluation forms consisted of two questions. We used one question to evaluate self-perceived knowledge of equipment and the other to evaluate anxiety around performing cystoscopy. The answer options encompassed a broad range in order to provide acceptable choices for learners at different levels. The first question asked learners to rate their knowledge of cystoscopy equipment on a 5-point Likert scale (1 = *no knowledge of equipment,* 5 = *can recognize equipment, put it together without assistance, and troubleshoot problems*). The second question asked participants to rate their level of anxiety with the procedure of cystoscopy on a 5-point Likert scale (1 = *high anxiety*, 5 = *no anxiety*). The postcourse evaluation forms consisted of the same two questions with the addition of a third question evaluating the overall utility of the activity. The third question also used a 5-point Likert scale (1 = *no knowledge/skill gained during course,* 5 = *superior knowledge/skill gained—I am ready to graduate and perform cysto on my own*). The postcourse evaluation form also prompted trainees to comment on whether any parts of the workshop were particularly helpful or were lacking in benefit. The full pre- and postcourse evaluation forms can be found in Appendix D. For the first two questions, pre- and postworkshop responses were compared using a paired *t* test.

In our most recent session, in September 2021, we tested participants' ability to identify and assemble cystoscopic

Copyright © 2022 Shah et al. This is an open-access publication distributed under the terms of the Creative Commons Attribution license.

equipment and perform cystoscopy immediately before and after the respective stations listed above using a checklist assessment. Proctors checked off boxes for successfully named equipment or completed tasks before and after the activity. This allowed us to compare the trainees' ability to identify equipment, assemble equipment, and perform the steps of cystoscopy independently both before and after they had completed the two stations in our workshop. The specific items evaluated can be seen in Appendix C. The averages of the total items correctly identified, the total number of pieces correctly assembled, and the total number of basic cystoscopy steps correctly performed pre- and postcourse were compared using a paired *t* test.

Results

Over the past 2 years, 27 residents participated in this activity. These residents ranged in level of training from PGY 1 to PGY 4. Of those residents, 20 (74%), completed both pre- and postcourse evaluation forms. Table 1 shows results from these evaluations. Before the activity, knowledge of cystoscopy equipment ranged from "I have no knowledge of equipment" to "I can recognize equipment and put it together without assistance," respectively 1 and 4 on the Likert scale that was used for evaluation. The mean precourse score for equipment familiarity was 3.1, and the postcourse mean increased to 4.4 ($p < .0001$), with all responding participants indicating that they could at least recognize equipment and assemble the cystoscope without assistance. In terms of anxiety level surrounding performing cystoscopy, initial responses from trainees ranged from some anxiety to no anxiety. The mean precourse Likert score for resident anxiety surrounding cystoscopy was 3.6; after the activity, the mean rose to 4.3 ($p < .0002$). Of the responding trainees, all indicated that they had either minimal or no anxiety after the workshop. On postcourse surveys, the mean score for overall course grade was 4.3 on the 5-point scale outlined above. All residents indicated that at least adequate knowledge and skill were gained from the course and that they felt ready to perform cystoscopy in a supervised setting.

Table 1. Mean Pre- and Postcourse Survey Results

Topic	Precourse Score	Postcourse Score	*p*
Knowledge of equipment[a]	3.1	4.4	<.0001
Anxiety with procedure[b]	3.6	4.3	<.0002
Overall course evaluation[c]		4.3	

[a]Rated on a 5-point Likert scale (1 = *no knowledge of equipment*, 5 = *can recognize equipment, put it together without assistance, and troubleshoot problems*).
[b]Rated on a 5-point Likert scale (1 = *high anxiety*, 5 = *no anxiety*).
[c]Rated on a 5-point Likert scale (1 = *no knowledge/skill gained during course*, 5 = *superior knowledge/skill gained—I am ready to graduate and perform cysto on my own*).

A compilation of responses to the open-ended questions at the end of our evaluation forms can be seen below. Generally, trainees found that having the opportunity to assemble and disassemble the equipment was a helpful exercise. Many participants left these open-ended questions blank, and some answers have not been included here to avoid redundancy.

- Any part of the course completely useless?
  - "None."
  - "No."
  - "Nope, it was great."
- Any part of the course that was absolutely essential?
  - "Hands on with patient instructors."
  - "Ability to practice as much as needed."
  - "Putting equipment together."
  - "Taking the cystoscope completely apart, putting it back together."
  - "Naming the equipment, assembling the cystoscope."
  - "Appreciated walking through steps of putting together the cystoscope. Often, I feel as though we are handed the cystoscope put together."
  - "Really liked the review of bladder findings as we do not get this education anywhere else."
  - "Video of pathology was high yield and instructive."
  - "Practicing with the pig bladder."
- Any suggestions for additions to the course?
  - "Review when to call urology to come to the OR."
  - "When to biopsy/consult urology."

In our most recent session in 2021, we assessed all 11 of the trainees participating in the course before and after each station (see Table 2). On average, residents were able to identify 3.6 out of eight pieces of cystoscopic equipment prior to completing the workshop. After completing the workshop, residents were able to correctly identify 7.7 out of eight pieces of cystoscopic equipment ($p < .0001$). Before the course, trainees were able to assemble on average 1.4 out of six pieces or cystoscopic equipment, while after the course, all residents were able to independently assemble all six pieces of cystoscopic equipment that were tested ($p < .0001$). Prior to the course, participants were able to complete, on average, 1.7 out of six steps of cystoscopy; afterward, they were able to complete an average

Table 2. Mean Pre- and Postcourse Checklist Assessment Results

Topic	Precourse Score	Postcourse Score	*p*
Identifying equipment (8 items)	3.6	7.7	<.0001
Assembling equipment (6 items)	1.4	6.0	<.0001
Simulated steps of cystoscopy performed (6 steps)	1.7	5.5	<.0001

Copyright © 2022 Shah et al. This is an open-access publication distributed under the terms of the Creative Commons Attribution license.

of 5.5 out of six without prompting ($p < .0001$). All residents showed improvement in ability to assemble the cystoscope and carry out the necessary steps of the procedure after completing the workshop.

Discussion

Since OB/GYN residents' exposure to cystoscopic procedures have the potential to be limited, we set out to develop a time-efficient course to boost cystoscopic skills for OB/GYN trainees. Specifically, we sought to increase their ability to identify and assemble equipment and to decrease their anxiety around performing the procedure using an hour-long workshop that focused on hands-on experience. We demonstrated decreased anxiety surrounding performing cystoscopy when comparing pre- and postcourse evaluations, as well as increased self-perceived knowledge of cystoscopic equipment. Furthermore, all residents who were assessed using the checklist in Appendix C improved their ability to assemble the equipment and their understanding of the steps of the procedure. This was accomplished by allowing residents to repeatedly practice identifying equipment, assembling equipment, and performing cystoscopy with direct supervision by a knowledgeable proctor. Residents were given immediate feedback and then allowed to retry stations numerous times, an educational technique known as rapid cycle deliberate practice that has previously been shown to be beneficial across diverse groups of learners.[7] In the comments section on evaluations, residents consistently stated that having time to assemble and disassemble the cystoscope repeatedly outside the operating room was valuable, as learners are often handed the scope in the operating room already assembled.

Our workshop does have limitations. The sample size is relatively small as data from only 20 participants were included, and only 11 participants were assessed objectively. The workshop represents the experience of a single institution with a single residency program and may not be as effective for other groups of residents at other programs. Our OB/GYN residents have simulation experiences about every 3 months and are accustomed to learning via simulation, which may have increased the utility of our exercise. The language in our pre- and postcourse evaluations concerning anxiety around performing cystoscopy may have caused some bias as anxiety can be a term with a negative association. Our data on self-perceived anxiety and knowledge of equipment, as well as the data we garnered using our checklist assessment, were all obtained immediately after the activity. It is possible that the residents may have had an inflated sense of how much comfort, skill, and knowledge were gained immediately after the workshop. It is also possible that some of the ability to identify and assemble cystoscopic equipment as well as to perform the steps of cystoscopy may atrophy with time. It would be beneficial to reassess residents with our checklist after a length of time has passed.

This activity has existed in some form since 2010 and has been improved upon since that time. Initially, the workshop began with an introductory lecture on cystoscopy. Participants found this lecture to be less useful than spending time working directly with cystoscopic equipment, so we removed the lecture from the course. We have found that it is important to use male pig bladders as some urethral length is necessary to secure the bladders and to provide a realistic simulation. The length of the urethral stump left on the pig bladder also has some importance as, if it is too long, it becomes difficult to secure and no longer mimics the female urethra.

In conclusion, our workshop provided an opportunity for OB/GYN residents to increase their ability to identify and assemble cystoscopic equipment and improved their ability to carry out the necessary steps of the procedure. The course was well received, and residents felt that it was helpful for easing anxiety around performing cystoscopy according to pre- and postcourse surveys.

Appendices

A. Instructors Guide.docx

B. Station Details.docx

C. Course Checklist.docx

D. Evaluation Forms.docx

All appendices are peer reviewed as integral parts of the Original Publication.

Nayan Shah, MD: Second-Year Resident, Department of Urology, Medical College of Wisconsin; ORCID: https://orcid.org/0000-0003-2368-9421

Robert Medairos, MD: Fifth-Year Resident, Department of Urology, Medical College of Wisconsin

Sumana Koduri, MD: Associate Professor, Department of Obstetrics and Gynecology, Medical College of Wisconsin

Carley Davis, MD: Associate Professor, Department of Urology, Medical College of Wisconsin

Disclosures
None to report.

Funding/Support
None to report.

Copyright © 2022 Shah et al. This is an open-access publication distributed under the terms of the Creative Commons Attribution license.

Ethical Approval

The Medical College of Wisconsin Institutional Review Board approved this project.

References

1. Review Committee for Obstetrics and Gynecology. *Case Log Information: Obstetrics and Gynecology*. Accreditation Council for Graduate Medical Education; 2021. Accessed February 21, 2021. https://www.acgme.org/Portals/0/PFAssets/ProgramResources/OBGYNCaseLogInfo.pdf?ver=2020-11-20-163905-143
2. Dune TJ, Blackwell RH, Griffin A, et al. Ready or not? Obstetrics and gynecology resident preparedness for female pelvic medicine and reconstructive surgery training. *Female Pelvic Med Reconstr Surg*. 2017;23(6):401-408. https://doi.org/10.1097/SPV.0000000000000418
3. Davis C, Mulligan M. Introduction to endoscopy for urology residents. *MedEdPORTAL*. 2015;11:10041. https://doi.org/10.15766/mep_2374-8265.10041
4. Bowling CB, Greer WJ, Bryant SA, et al. Testing and validation of a low-cost cystoscopy teaching model: a randomized controlled trial. *Obstet Gynecol*. 2010;116(1):85-91. https://doi.org/10.1097/AOG.0b013e3181e45a52
5. Do L, Pasha K, Sanchez S, Montoya TI, Maldonado PA. A structured, hybrid cystoscopy simulation curriculum for obstetrics/gynecology residents. *Female Pelvic Med Reconstr Surg*. 2021;27(10):637-641. https://doi.org/10.1097/SPV.0000000000001018
6. Lenherr SM, Crosby EC, Cameron AP. Cystoscopic findings: a video tutorial. *Int Urogynecol J*. 2015;26(6):921-923. https://doi.org/10.1007/s00192-014-2614-4
7. Peng CR, Schertzer K. Rapid cycle deliberate practice in medical simulation. *StatPearls*. Updated July 26, 2021. Accessed December 1, 2021. https://www.statpearls.com/ArticleLibrary/viewarticle/63853

Received: June 18, 2021
Accepted: November 6, 2021
Published: February 7, 2022

Copyright © 2022 Shah et al. This is an open-access publication distributed under the terms of the Creative Commons Attribution license.
